# Automated plant species identification—Trends and future directions

**DOI:** 10.1371/journal.pcbi.1005993

**Published:** 2018-04-05

**Authors:** Jana Wäldchen, Michael Rzanny, Marco Seeland, Patrick Mäder

**Affiliations:** 1 Department of Biogeochemical Integration, Max Planck Institute for Biogeochemistry, Jena, Thuringia, Germany; 2 Software Engineering for Safety-Critical Systems Group, Technische Universität Ilmenau, Ilmenau, Thuringia, Germany; University of Georgia Warnell School of Forestry and Natural Resources, UNITED STATES

## Abstract

Current rates of species loss triggered numerous attempts to protect and conserve biodiversity. Species conservation, however, requires species identification skills, a competence obtained through intensive training and experience. Field researchers, land managers, educators, civil servants, and the interested public would greatly benefit from accessible, up-to-date tools automating the process of species identification. Currently, relevant technologies, such as digital cameras, mobile devices, and remote access to databases, are ubiquitously available, accompanied by significant advances in image processing and pattern recognition. The idea of automated species identification is approaching reality. We review the technical status quo on computer vision approaches for plant species identification, highlight the main research challenges to overcome in providing applicable tools, and conclude with a discussion of open and future research thrusts.

## Author summary

Plant identification is not exclusively the job of botanists and plant ecologists. It is required or useful for large parts of society, from professionals (such as landscape architects, foresters, farmers, conservationists, and biologists) to the general public (like ecotourists, hikers, and nature lovers). But the identification of plants by conventional means is difficult, time consuming, and (due to the use of specific botanical terms) frustrating for novices. This creates a hard-to-overcome hurdle for novices interested in acquiring species knowledge. In recent years, computer science research, especially image processing and pattern recognition techniques, have been introduced into plant taxonomy to eventually make up for the deficiency in people's identification abilities. We review the technical status quo on computer vision approaches for plant species identification, highlight the main research challenges to overcome in providing applicable tools, and conclude with a discussion of open and future research thrusts.

## Introduction

One of the most obvious features of organic life is its remarkable diversity [1]. Despite the variation of organisms, a more experienced eye soon discerns that organisms can be grouped into taxa. Biology defines taxa as formal classes of living things consisting of the taxon's name and its description [2]. The assignment of an unknown living thing to a taxon is called identification [3]. This article specifically focuses on plant identification, which is the process of assigning an individual plant to a taxon based on the resemblance of discriminatory and morphological plant characters, ultimately arriving at a species or infraspecific name. These underlying characters can be qualitative or quantitative. Quantitative characters are features that can be counted or measured, such as plant height, flower width, or the number of petals per flower. Qualitative characters are features such as leaf shape, flower color, or ovary position. Individuals of the same species share a combination of relevant identification features. Since no two plants look exactly the same, it requires a certain degree of generalization to assign individuals to species (or, in other words, assign objects to a fuzzy prototype).

The world inherits a very large number of plant species. Current estimates of flowering plant species (angiosperms) range between 220,000 [4, 5] and 420,000 [6]. Given the average 20,000 word vocabulary of an educated native English speaker, even teaching and learning the "taxon vocabulary" of a restricted region becomes a long-term endeavor [7]. In addition to the complexity of the task itself, taxonomic information is often captured in languages and formats hard to understand without specialized knowledge. As a consequence, taxonomic knowledge and plant identification skills are restricted to a limited number of persons today.

The dilemma is exacerbated since accurate plant identification is essential for ecological monitoring and thereby especially for biodiversity conservation [8, 9]. Many activities, such as studying the biodiversity of a region, monitoring populations of endangered species, determining the impact of climate change on species distribution, payment of environmental services, and weed control actions are dependent upon accurate identification skills [8, 10]. With the continuous loss of biodiversity [11], the demand for routine species identification is likely to further increase, while at the same time, the number of experienced experts is limited and declining [12].

Taxonomists are asking for more efficient methods to meet identification requirements. More than 10 years ago, Gaston and O’Neill [13] argued that developments in artificial intelligence and digital image processing will make automatic species identification based on digital images tangible in the near future. The rich development and ubiquity of relevant information technologies, such as digital cameras and portable devices, has brought these ideas closer to reality. Furthermore, considerable research in the field of computer vision and machine learning resulted in a plethora of papers developing and comparing methods for automated plant identification [14–17]. Recently, deep learning convolutional neural networks (CNNs) have seen a significant breakthrough in machine learning, especially in the field of visual object categorization. The latest studies on plant identification utilize these techniques and achieve significant improvements over methods developed in the decade before [18–23].

Given these radical changes in technology and methodology and the increasing demand for automated identification, it is time to analyze and discuss the status quo of a decade of research and to outline further research directions. In this article, we briefly review the workflow of applied machine learning techniques, discuss challenges of image based plant identification, elaborate on the importance of different plant organs and characters in the identification process, and highlight future research thrusts.

## Machine learning for species identification

**Fig 1:**
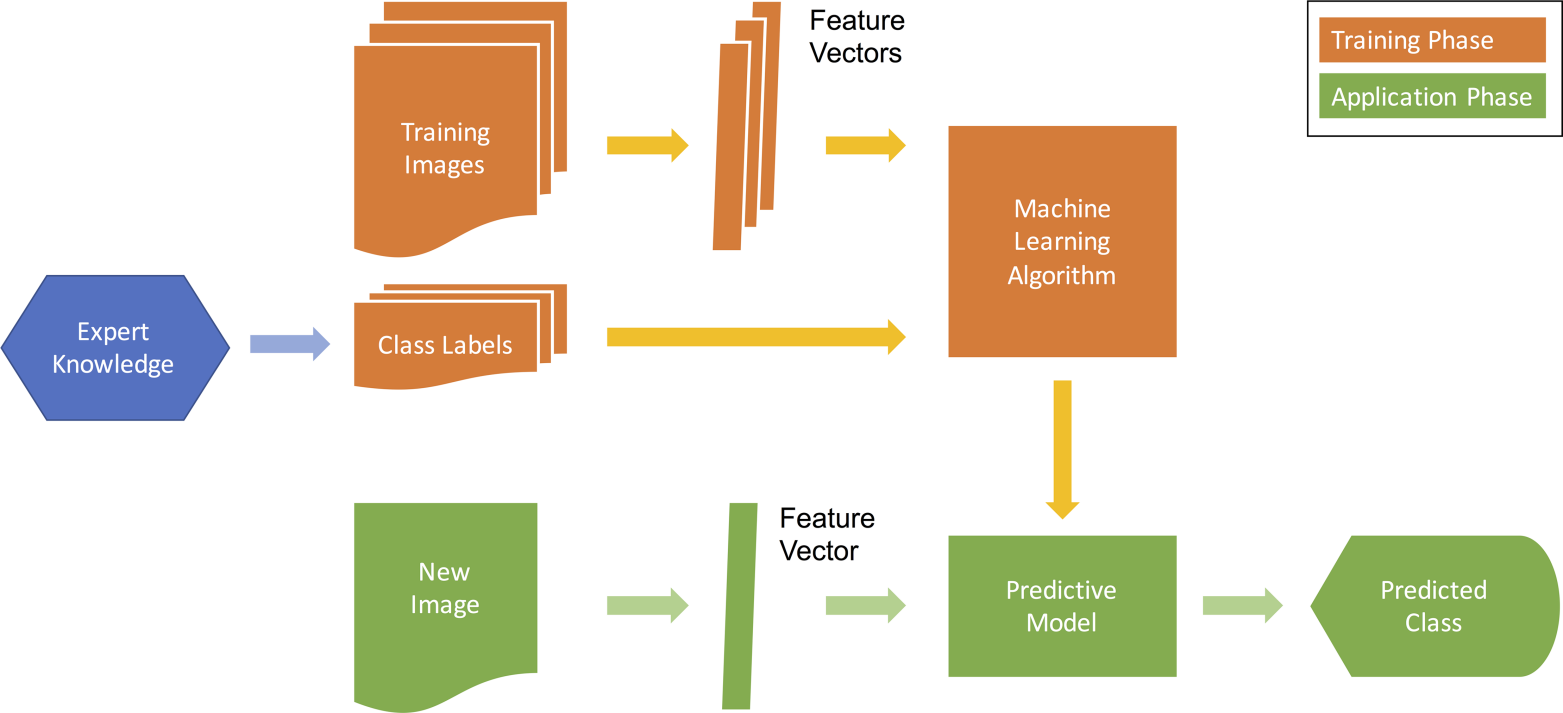
Fundamental steps of supervised machine learning for image-based species identification.

From a machine learning perspective, plant identification is a supervised classification problem, as outlined in Fig 1. Solutions and algorithms for such identification problems are manifold and were comprehensively surveyed by Wäldchen and Mäder [16] and Cope et al. [17]. The majority of these methods are not applicable right away but rather require a training phase in which the classifier learns to distinguish classes of interest. For species identification, the training phase (orange in Fig 1) comprises the analysis of images that have been independently and accurately identified as taxa and are now used to determine a classifier's parameters for providing maximum discrimination between these trained taxa. In the application phase (green in Fig 1), the trained classifier is then exposed to new images depicting unidentified specimens and is supposed to assign them to one of the trained taxa.

Images are usually composed of millions of pixels with associated color information. This information is too extensive and cluttered to be directly used by a machine learning algorithm. The high dimensionality of these images is therefore reduced by computing feature vectors, i.e., a quantified representation of the image that contains the relevant information for the classification problem. During the last decade, research on automated species identification mostly focused on the development of feature detection, extraction, and encoding methods for computing characteristic feature vectors. Initially, designing and orchestrating such methods was a problem-specific task, resulting in a model customized to the specific application, e.g., the studied plant parts like leaves or flowers. For example, Wu et al. [24] employ a processing chain comprised of image binarization to separate background and the leaf, image denoising, contour detection, and eventually extracting geometrical derivations of 12 leaf shape features. The approach was evaluated on 32 species and delivered an identification accuracy of 90%. However, this approach could only deal with species differing largely in their leaf shapes. Jin et al. [25] propose leaf tooth features extracted after binarization, segmentation, contour detection, and contour corner detection. The proposed method achieved an average classification rate of around 76% for the eight studied species but is not applicable to species with no significant appearances of leaf teeth [19]. The sole step from an image to a feature vector, however, typically required about 90% of the development time and extensive expert knowledge [19].

Model-free approaches aim to overcome the described limitations of model-based approaches. They do not employ application-specific knowledge and therefore promise a higher degree of generalization across different classes, i.e., species and their organs. The core concept of model-free approaches is the detection of characteristic interest points and their description using generic algorithms, such as scale-invariant feature transform (SIFT), speeded-up robust features (SURF), and histogram of gradients (HOG). These descriptors capture visual information in a patch around each interest point as orientation of gradients and have been successfully used for manifold plant classification studies, e.g., [26–28]. Seeland et al. [29] comparatively evaluate alternative parts of a model-free image classification pipeline for plant species identification. They found the SURF detector in combination with the SIFT local shape descriptor to be superior over other detector–descriptor combinations. For encoding interest points, in order to form an characteristic image descriptor for classification, they found the Fisher Kernel encoding to be superior.

**Fig 2:**
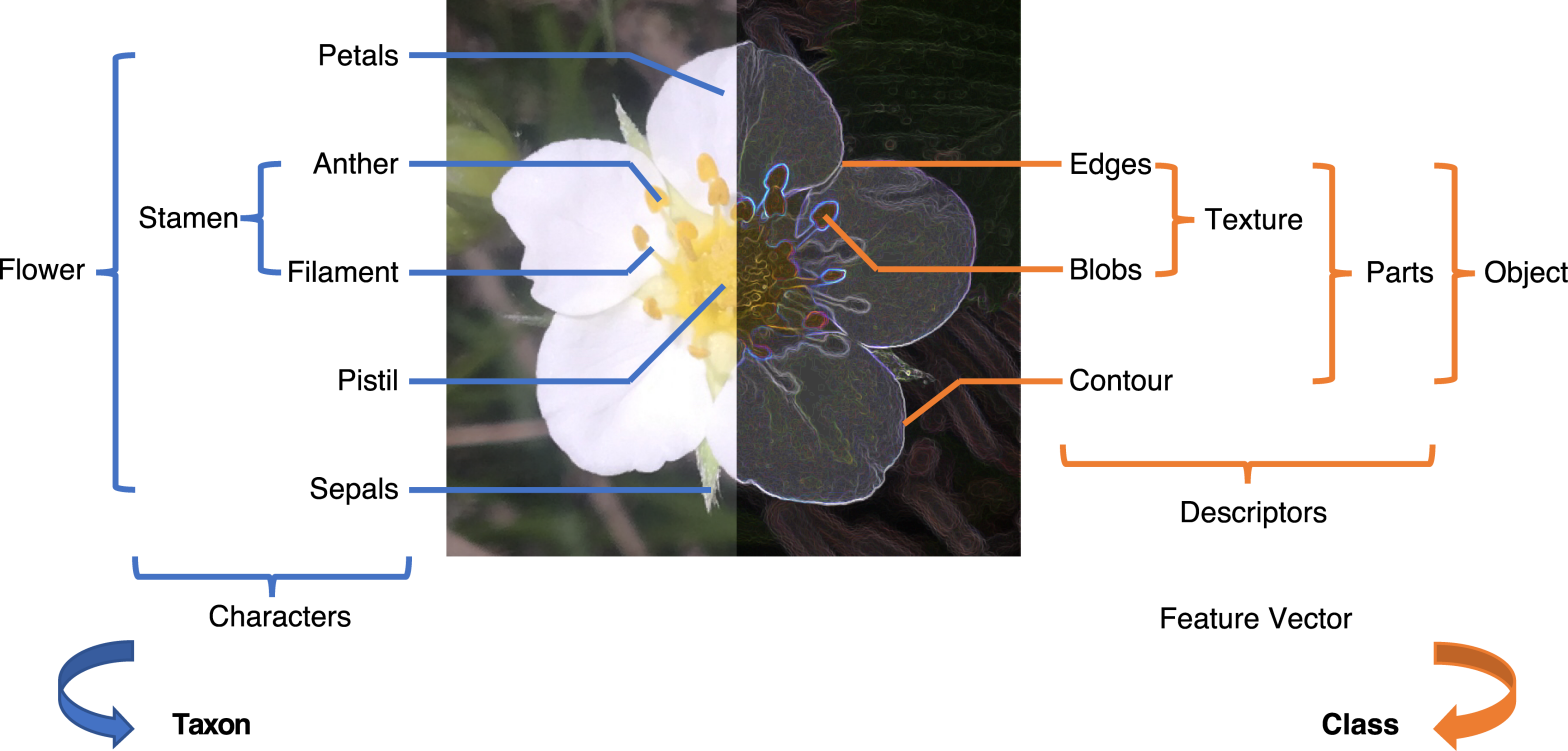
Botanists' (left) versus computer vision (right) description of flowers.

The next obvious step in automated plant species identification and many other machine learning problems was removing an explicit decision about features to be described entirely. In the last years, deep learning CNNs have seen a significant breakthrough in computer vision due to the availability of efficient and massively parallel computing on graphics processing units (GPUs) and the availability of large-scale image data necessary for training deep CNNs with millions of parameters [19]. In contrast to model-based and model-free techniques, CNNs do not require explicit and hand-crafted feature detection and extraction steps. Instead, both become part of the iterative training process, which automatically discovers a statistically suitable image representation (similar to a feature vector) for a given problem. The fundamental concept of deep learning is a hierarchical image representation composed of building blocks with increasing complexity per layer. In a similar way, nature is compositional, i.e., small units form larger units, and each aggregation level increases the diversity of the resulting structure (Fig 2). Such hierarchical representations achieve classification performances that were mostly unachievable using shallow learning methods with or without hand-crafted features (see Table 1).

**Table 1:** Increasing classification accuracy achieved with evolving machine learning approaches on popular plant species benchmark datasets.

	Model-based approach		Model-free approach		Deep learning	
Dataset	Accuracy	Author	Accuracy	Author	Accuracy	Author
Swedish leaf	82.0%	[30]	93.7%	[26]	99.8%	[18]
Flavia	90.3%	[24]	95.9%	[27]	99.7%	[31]
Leafsnap	73.0%	[14]	72.6%	[20]	97.6%	[20]
ICL	83.8%	[32]	91.3%	[32]	93.9%	[21]
Oxford Flower 17	-	-	91.8%	[29]	96.6%	[22]
Oxford Flower 102	-	-	90.2%	[28]	96.6%	[23]

## Challenges in image-based taxa identification

In providing a reliable and applicable automated species identification process, researchers need to consider the following main challenges: (a) a vast number of taxa to be discriminated from one another; (b) individuals of the same species that vary hugely in their morphology; (c) different species that are extremely similar to one another; (d) specimen or other objects that are not covered by the trained classifier; and (e) large variation induced by the image acquisition process in the field.

### Large number of taxa to be discriminated

The world exhibits a very large number of plant species. Distinguishing between a large number of classes is inherently more complex than distinguishing between just a few and typically requires substantially more training data to achieve satisfactory classification performance. Even when restricting the focus to the flora of a region, thousands of species need to be supported. For example, the flora of the German state of Thuringia exhibits about 1,600 flowering species [33]. Similarly, when restricting the focus to a single genus, this may still contain many species, e.g., the flowering plant genus *Dioscorea* aggregates over 600 species [17]. Only a few studies with such large numbers of categories have been conducted so far. For example, the important "ImageNet Large Scale Visual Recognition Challenge 2017" involves 1,000 categories that cover a wide variety of objects, animals, scenes, and even some abstract geometric concepts such as a hook or a spiral [34].

### Large intraspecific visual variation

**Fig 3:**
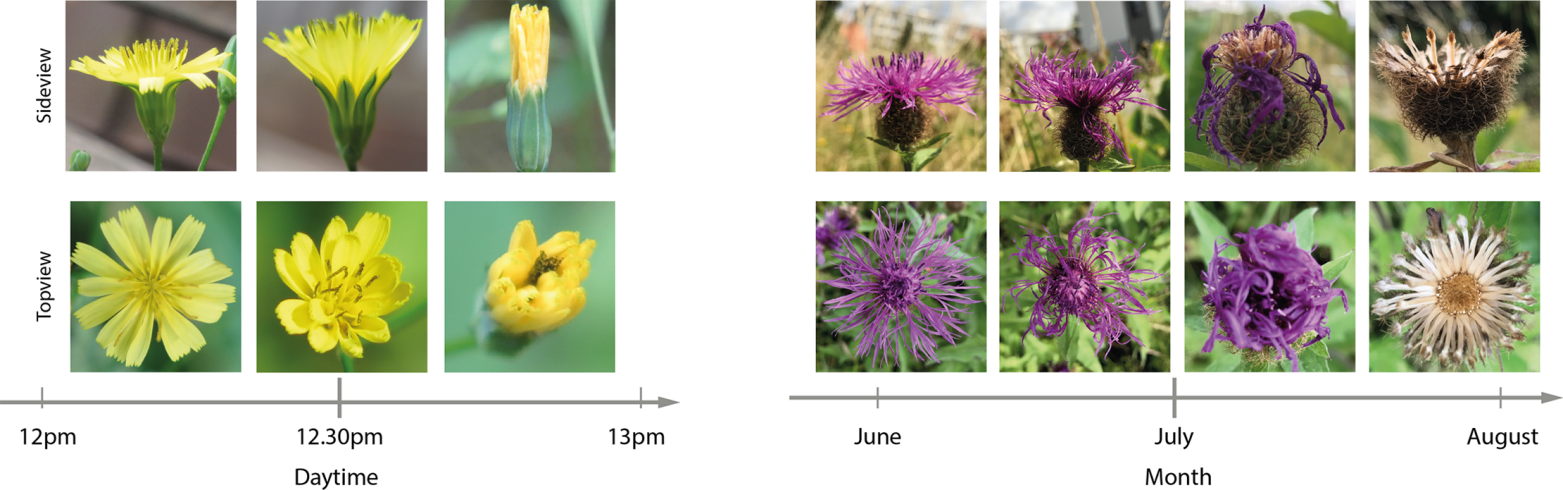
Visual variation of *Lapsana communis*'s flower throughout the day from two perspectives (left) and visual variation of *Centaurea pseudophrygia*'s flower throughout the season and flowering stage (right).

Plants belonging to the same species may show considerable differences in their morphological characteristics depending on their geographical location and different abiotic factors (e.g., moisture, nutrition, and light condition), their development stage (e.g., differences between a seedling and a fully developed plant), the season (e.g., early flowering stage to a withered flower), and the daytime (e.g., the flower is opening and closing during the day). These changes in morphological characteristics can occur on the scale of individual leaves (e.g., area, width, length, shape, orientation, and thickness), flowers (e.g., size, shape, and color), and fruits but may also affect the entire plant. Examples of visual differences of flowers during the daytime and the season are given in Fig 3. In addition to the spatial and temporal variation, the shape of leaves and flowers may vary continuously or discretely along a single individual. For example, the leaf shape of field scabious (*Knautia arvensis*), a common plant in grassy places, ranges from large entire or dentate lanceolate ground leafs over deeply lobed and almost pinnate stem leafs to small and again lanceolate and entire upper stem leafs. Furthermore, diseases commonly affect the surface of leaves, ranging from discoloration to distinct marking, while insects often alter a leaf's shape by consuming parts of it. Some of this variation is systematic, particularly the allometric scaling of many features, but much variation is also idiosyncratic, reflecting the expression of individual genotypic and phenotypic variation related to the factors mentioned.

### Small interspecific visual variation

Closely related species may be extremely similar to one another. Even experienced botanists are challenged to safely distinguish species that can be identified only by almost invisible characteristics [35]. Detailed patterns in the form of particular morphological structures may be crucial and may not always be readily captured, e.g., in images of specimens. For example, the presence of flowers and fruits is often required for an accurate discrimination between species with high interspecific similarity, but these important characteristics are not present during the whole flowering season and therefore are missing in many images. Furthermore, particular morphological structures which are crucial for discrimination may not be captured in an image of a specimen, even when the particular organ is visible (e.g., the number of stamens or ovary position in the flower).

### Rejecting untrained taxa

An automated taxon identification approach not only needs to be able to match an individual specimen to one of the known taxa, but should also be able to reject specimens that belong to a taxon that was not part of the training set. In order to reject unknown taxa, the classification method could produce low classification scores across all known classes for "new" taxa. However, aiming for a classifier with such characteristics conflicts with the goal of tolerating large intraspecific variation in classifying taxa. Finding a trade-off between sensitivity and specificity is a particular challenge in classifier design and training.

### Variation induced by the acquisition process

Further variation is added to the images through the acquisition process itself. Living plants represent 3D objects, while images capture 2D projections, resulting in potentially large differences in shape and appearance, depending on the perspective from which the image is taken. Furthermore, image-capturing typically occurs in the field with limited control of external conditions, such as illumination, focus, zoom, resolution, and the image sensor itself [2]. These variations are especially relevant for an automated approach in contrast to human perception.

## Status quo

In the last decade, research in computer vision and machine learning has stimulated manifold methods for automated plant identification. Existing image-based plant identification approaches differ in three main aspects: (a) the analyzed plant organs, (b) the analyzed organ characters, and (c) the complexity of analyzed images. An extensive overview of studied methods is given by Wäldchen and Mäder [16] and is briefly summarized below.

### Relevant organs for automated identification

Above the ground, plants may be composed of four visible organ types: stem, leaf, flower, and fruit. In a traditional identification process, people typically consider the plant as a whole, but also the characteristics of one or more of these organs to distinguish between taxa. In case of automated identification, organ characteristics were analyzed separately, too. For the following reasons one image alone is typically not sufficient: (a) organs may differ in scale and cannot be depicted in detail along with the whole plant or other organs; and (b) different organs require different optimal image perspectives (e.g., leaves are most descriptive from the top, while the stem is better depicted from the side).

A majority of previous studies solely utilized the **leaf** for discrimination [16]. The reason is a more methodological one, rather than meaning that leaves are a more discriminative part of plants from a botanical perspective. On the contrary, manual identification of plants in the vegetative state is considered much more challenging than in the flowering state. From a computer vision perspective, leaves have several advantages over other plant morphological structures, such as flowers, stems, or fruits. Leaves are available for examination throughout most of the year. They can easily be collected, preserved, and imaged due to their planar geometric properties. These aspects simplify the data acquisition process [17] and have made leaves the dominantly studied plant organ for automated identification methods in the past. In situ top-side leaf images in front of a natural background were shown to be the most effective nondestructive type of image acquisition [36]. Leaves usually refer only to broad leaves, while needles were neglected or treated separately.

Often, the visually most prominent and perceivable part of a plant is its **flower**. Traditional identification keys intensively refer to flowers and their parts for determination. In contrast, previous studies on automated identification rarely used flowers for discrimination [16]. Typically, flowers are only available during the blooming season, i.e., a short period of the year. Due to being complex 3D objects, there is a considerable number of variations in viewpoint, occlusions, and scale of flower images compared to leaf images. If captured in their habitat, images of flowers vary due to lighting conditions, time, date, and weather. All these aspects make flower-based classification a challenging task. However, accurate, automated identification supporting a realistic number of taxa will hardly be successful without the analysis of flowers.

Towards a more mature automated identification approach, solely analyzing one organ will often not be sufficient, especially when considering all the challenges discussed in the previous section. Therefore, more recent research started exploring **multi-organ-based plant identification**. The Cross Language Evaluation Forum (ImageCLEF) conference has organized a challenge dedicated to plant identification since 2011. The challenge is described as plant species retrieval based on multi-image plant observation queries and is accompanied by a dataset containing different organs of plants since 2014. Participating in the challenge, Joly et al. [37] proposed a multiview approach that analyzes up to five images of a plant in order to identify a species. This multiview approach allows classification at any period of the year, as opposed to purely leaf-based or flower-based approaches that rely on the supported organ to be visible. Initial experiments demonstrate that classification accuracy benefits from the complementarities of the different views, especially in discriminating ambiguous taxa [37]. A considerable burden in exploring this research direction is acquiring the necessary training data. However, by using mobile devices and customized apps (e.g., Pl@ntNet [38], Flora Capture [39]), it is possible to quickly capture multiple images of the same plant observed at the same time, by the same person, and with the same device. Each image, being part of such an observation, can be labeled with contextual metadata, such as the displayed organ (e.g., plant, branch, leaf, fruit, flower, or stem), time and date, and geolocation, as well as the observer.

It is beneficial if training images cover a large variety of scenarios, i.e., different organs from multiple perspective and at varying scale. This helps the model to learn adequate representations under varying circumstances. Furthermore, images of the same organ acquired from different perspectives often contain complementary visual information, improving accuracy in observation-based identification using multiple images. A **structured observation** approach with well defined image conditions (e.g., Flora Capture) is beneficial for finding a balance between a tedious observation process acquiring every possible scenario and a superficial acquisition that misses the characteristic images required for training.

### Relevant characters for automated identification

A plant and its organs (i.e., objects in computer vision) can be described by various characters, such as color, shape, growing position, inflorescence of flowers, margin, pattern, texture, and vein structure of the leaves. These characters are extensively used for traditional identification, with many of them also being studied for automated identification. Previous research proposed numerous methods for describing general as well as domain-specific characteristics. Extensive overviews of the utilized characteristics, as well as of the methods used for capturing them in a formal description, are given by Wäldchen and Mäder [16] and Cope et al. [17].

**Leaf shape** is the most studied characteristic for plant identification. A plethora of methods for its description can be found in previous work [16, 17]. Also, most traditional taxonomic keys involve leaf shape for discrimination, the reason being that, although species' leaf shape differs in detail, general shape types can easily be distinguished by people. However, while traditional identification categorizes leaf shape into classes (e.g., ovate, oblique, oblanceolate), computerized shape descriptors either analyze the contour or the whole region of a leaf. Initially, basic geometric descriptors, such as aspect ratio, rectangularity, circularity, and eccentricity, were used to describe a shape. Later, more sophisticated descriptions, such as center contour distance, Fourier descriptors, and invariant moments, were intensively studied [16, 17].

In addition to the shape characteristic, various researchers also studied **leaf texture**, described by methods like Gabor filters, gray-level co-occurrence matrices (GLCM), and fractal dimensions [40–42]. Although texture is often overshadowed by shape as the dominant or more discriminative feature for leaf classification, it has been demonstrated to be of high discriminative power and complementary to shape information [16, 43]. In particular, leaf texture captures leaf venation information as well as any eventual directional characteristics, and more generally allows describing fine nuances or micro-texture at the leaf surface [44]. Furthermore, leaf texture analysis allows to classify a plant by having only a portion of a leaf available without depending, e.g., on the shape of the full leaf or its color. Therefore, texture analysis can be beneficial for botanists and researchers that aim to identify damaged plants.

The **vein structure** as a leaf-specific characteristic also played a subordinate role in previous studies. Venation extraction is not trivial, mainly due to a possible low contrast between the venation and the rest of the leaf blade structure [45]. Some authors have simplified the task by using special equipment and treatments that render images with more clearly identified veins (mainly chemical leaf clarification) [45, 46]. However, this defeats the goal of having users get an automated identification for specimens that they have photographed with ordinary digital cameras.

**Leaf color** is considered a less discriminative character than shape and texture. Leaves are mostly colored in some shade of green that varies greatly under different illumination [44], creating a low interclass color variability. In addition, there is high intraclass variability. For example, the leaves belonging to the same species or even the same plant can present a wide range of colors depending on the season and the plant's overall condition (e.g., nutrients and water). Regardless of the aforementioned complications, color may still contribute to plant identification, e.g., for considering leaves that exhibit an extraordinary hue [44]. However, further investigation on the leaf color character is required.

While the shape of the leaves is of very high relevance, **flower shape** has hardly been considered so far. Interestingly, flower shape is an important characteristic in the traditional identification process. It is dividing plants into families and genera and is thereby considerably narrowing the search space for identification. However, previous attempts for describing flower shape in a computable form did not find it to be very discriminative [47]. A major reason is the complex 3D structure of flowers, which makes its shape vary depending on the perspective from which an image was taken. Furthermore, flower petals are often soft and flexible, which is making them bend, curl or twist and letting the shape of the same flower appear very differently. A flower's shape also changes throughout the season [29] and with its age to the extent where petals even fall off [48], as visualized in Fig 3.

**Flower color** is a more discriminative character [48, 49]. Many traditional field guides divide plants into groups according to their flower color. For automated identification, color has been mostly described by color moments and color histograms [16]. Due to the low dimensionality and the low computational complexity of these descriptors, they are also suitable for real-time applications. However, solely analyzing color characters, without, e.g., considering flower shape, cannot classify flowers effectively [48, 49]. Flowers are often transparent to some degree, i.e., the perceived color of a flower differs depending on whether the light comes from the back or the front of the flower. Since flower images are taken under different environmental conditions, the variation in illumination is greatly affecting analysis results. This motivated the beneficial usage of photometric invariant color characters [29, 50].

Various previous studies showed that no single character may be sufficient to separate all desired taxa, making character selection and description a challenging problem. For example, whilst leaf shape may be sufficient to distinguish some taxa, others may look very similar to each other but have differently colored leaves or texture patterns. The same applies to flowers, where specimens of the same color may differ in their shape or texture. Therefore, various studies do not only consider one type of character but use a **combination of characteristics** for describing leaves and flowers [16]. The selection of characteristics is always specific for a certain set of taxa and might not be applicable to others. Meaningful characters for, e.g., flower shape can only be derived if there are flowers of sufficient size and potentially flat structure. The same applies to leaf shape and texture. This reflects a fundamental drawback of shallow learning methods using hand-crafted features for specific characters.

### Deep learning

Deep artificial neural networks automate the critical feature extraction step by learning a suitable representation of the training data and by systematically developing a robust classification model. Since about 2010, extensive studies with folded neural networks have been conducted on various computer vision problems. In 2012, for the first time a deep learning network architecture with eight layers (AlexNet) won the prestigious ImageNet Challenge (ILSVRC) [51]. In the following years, the winning architectures grew in depth and provided more sophisticated mechanisms that centered around the design of layers, the skipping of connections, and on improving gradient flow. In 2015, ResNet [52] won ILSVRC with a 152 layer architecture and reached a top-5 classification error of 3.6%, being better than human performance (5.1%) [34]. As for many object classification problems, CNNs produce promising and constantly improving results on automated plant species identification. One of the first studies on plant identification utilizing CNNs is Lee et al.'s [53, 54] leaf classifier that uses the AlexNet architecture pretrained on the ILSVRC2012 dataset and reached an average accuracy of 99.5% on a dataset covering 44 species. Zhang et al. [55] used a six-layer CNN to classify the Flavia dataset and obtained an accuracy of 94,69%. Barre et al. [19] further improved this result by using a 17-layer CNN and obtained an accuracy of 97.9%. Eventually, Sun et al. [31] study the ResNet architecture and found a 26-layer network to reach best performance with 99.65% on the Flavia dataset. Simon et al. [56] used CNNs (AlexNet and VGG19) for feature detection and extraction inside a part constellation modeling framework. Using Support Vector Machine (SVM) as classifier, they achieved 95.34% on the Oxford Flowers 102 dataset. Table 1 contrasts the best previously reported classification results of model-based, model-free and CNN-based approaches on benchmark plant image datasets. A comparison shows that CNN classification performance was unachievable using traditional and shallow learning approaches.

### Training data and benchmarks

Merely half of the previous studies on automated plant identification evaluated the proposed method with established benchmark datasets allowing for replication of studies and comparison of methods (see Table 2). The other half solely used proprietary leaf image datasets not available to the public [16].

**Table 2:** Overview of previously studied benchmark datasets.

Dataset	Author	# Species	# Images	Acquisition	Background	Organs	Life form
Swedish leaf	[30]	15	1,125	scan	plain	leaves	trees
Flavia	[24]	32	1,907	scan + photo	plain	leaves	trees
Leafsnap	[58]	185	30,866	scan + photo	plain	leaves	trees
ICL	[59]	220	17,032	scan + photo	plain	leaves	herb, tree
Oxford Flower 17	[48]	17	1,360	photo	natural	flower	herbs
Jena Flower 30	[29]	30	1,479	photo	natural	flower	herbs
Oxford Flower 102	[49]	102	8,189	photo	natural	flower	herbs
PlantCLEF16	[60]	1,000	113,205	photo	natural	fruit, flower, leaves, stem	herb, tree, fern

The images contained in these datasets (proprietary as well as benchmark) fall into three categories: scans, pseudo-scans, and photos. While scan and pseudo-scan categories correspond respectively to leaf images obtained through scanning and photography in front of a simple background, the photo category corresponds to leaves or flowers photographed on natural background. The majority of utilized leaf images are scans and pseudo-scans [16]. Typically fresh material, i.e., simple, healthy, and not degraded leaves, were collected and imaged in the lab. This fact is interesting since it considerably simplifies the classification task. If the object of interest is imaged against a plain background, the often necessary segmentation for distinguishing foreground and background can be performed in a fully automated way with high accuracy.

Leaves imaged in the natural environment, as well as degraded leaves largely existing in nature, such as deformed, partial, overlapped, and compounded leaves (leaves consisting of two or more leaflets born on the same leafstalk), are largely avoided in the current studies. Segmenting the leaf with natural background is particularly difficult when the background shows a significant amount of overlapping, almost unicolor elements. This is often unavoidable when imaging leaves in their habitat. Interferences around the target leaves, such as small stones and ruderals may create confusion between the boundaries of adjacent leaves. Compound leaves are particularly difficult to recognize and existing studies that are designed for the recognition of simple leaves can hardly be applied directly to compound leaves. This is backed up by the variation of a compound leaf—it is not only caused by morphological differences of leaflets, but also by changes in the leaflet number and arrangements [57].

The lower part of Table 2 shows benchmark datasets containing flower images. The images of the Oxford Flower 17 and 102 datasets have been acquired by searching the internet and by selecting images of species with substantial variation in shape, scale, and viewpoint. The PlantCLEF2015/2016 dataset consists of images with different plant organs or plant views (i.e., entire plant, fruit, leaf, flower, stem, branch, and leaf scan). These images were submitted by a variety of users of the mobile Pl@ntNet application. The recently published Jena Flower 30 dataset [29] contains images acquired in the field as top-view flower images using an Apple iPhone 6 throughout an entire flowering season. All images of these flower benchmark datasets are photos taken in the natural environment.

### Applicable identification tools

Despite intensive and elaborate research on automated plant species identification, only very few studies resulted in approaches that can be used by the general public, such as Leafsnap [61] and Pl@ntNet [37]. Leafsnap, developed by researchers from Columbia University, the University of Maryland, and the Smithsonian Institution, was the first widely distributed electronic field guide. Implemented as a mobile app, it uses computer vision techniques for identifying tree species of North America from photographs of their leaves on plain background. The app retrieves photos of leaves similar to the one in question. However, it is up to the user to make the final decision on what species matches the unknown one. LeafSnap achieves a top-1 recognition rate of about 73% and a top-5 recognition rate of 96.8% for 184 tree species [61]. The app has attracted a considerable number of downloads but has also received many critical user reviews [62] due to its inability to deal with cluttered backgrounds and within-class variance.

Pl@ntNet is an image retrieval and sharing application for the identification of plants. It is being developed in a collaboration of four French research organizations (French agricultural research and international cooperation organization [Cirad], French National Institute for Agricultural Research [INRA], French Institute for Research in Computer Science and Automation [Inria], and French National Research Institute for Sustainable Development [IRD]) and the Tela Botanica network. It offers three front-ends, an Android app, an iOS app, and a web interface, each allowing users to submit one or several pictures of a plant in order to get a list of the most likely species in return. The application is becoming more and more popular. The application has been downloaded by more than 3 million users in about 170 countries. It was initially restricted to a fraction of the European flora (in 2013) and has since been extended to the Indian Ocean and South American flora (in 2015) and the North African flora (in 2016). Since June 2015, Pl@ntNet applies deep learning techniques for image classification. The network is pretrained on the ImageNet dataset and periodically fine-tuned on steadily growing Pl@ntNet data. Joly et al. [63] evaluated the Pl@ntNet application, which supported the identification of 2,200 species at that time, and reported a 69% top-5 identification rate for single images. We could not find published evaluation results on the current performance of the image-based identification engine. However, reviews request better accuracy [15]. We conclude that computer vision solutions are still far from replacing the botanist in extracting plant characteristic information for identification. Improving the identification performance in any possible way remains an essential objective for future research. The following sections summarize important current research directions.

## Open problems and future directions

### Utilizing latest machine learning developments

While the ResNet architecture is still state-of-the-art, evolutions are continuously being proposed, (e.g., [64]). Other researchers work on alternative architectures like ultra-deep (FractalNet) [65] and densely connected (DenseNet) [66] networks. These architectures have not yet been evaluated for plant species identification. New architectures and algorithms typically aim for higher classification accuracy, which is clearly a major goal for species identification; however, there are also interesting advances in reducing the substantial computational effort and footprint of CNN classifiers. For example, SqueezeNet [67] achieves accuracy comparable to AlexNet but with 50 times fewer parameters and a model that is 510 times smaller. Especially when aiming for identification systems that run on mobile devices, these developments are highly relevant and should be evaluated in this context.

Current studies still mostly operate on the small and nonrepresentative datasets used in the past. Only a few studies train CNN classifiers on large plant image datasets, demonstrating their applicability in automated plant species identification systems [68]. Given the typically "small" amounts of available training data and the computational effort for training a CNN, transfer learning has become an accepted procedure (meaning that a classifier will be pretrained on a large dataset, e.g., ImageNet, before the actual training begins). The classifier will then only be fine-tuned to the specific classification problem by training of a small number of high-level network layers proportional to the amount of available problem-specific training data. Researchers argue that this method is superior for problems with ≤ 1 M training images. Most previous studies on plant species identification utilized transfer learning, (e.g., [54, 69]). Once a sufficiently large plant dataset has been acquired, it would be interesting to compare current classification results with those of a plant identification CNN solely trained on images depicting plant taxa.

Another approach tackling the issue of small datasets is using data augmentation schemes, commonly including simple modifications of images, such as rotation, translation, flipping, and scaling. Using augmentation for improving the training process has become a standard procedure in computer vision. However, the diversity that can be reached with traditional augmentation schemes is relatively small. This motivates the use of synthetic data samples, introducing more variability and enriching the dataset, in order to improve the training process. A promising approach in this regard are Generative Adversarial Networks (GANs) that are able to generate high-quality, realistic, natural images [70].

Without the complicated and time-consuming process for designing an image analysis pipeline, deep learning approaches can also be applied by domain experts directly, i.e., botanists and biologists with only a basic understanding of the underlying machine learning concepts. Large-scale organizations provide a competing and continuously improving set of openly available machine learning frameworks, such as Caffe2, MXNet, PyTorch, and TensorFlow. Developments like Keras specifically target newcomers in machine learning and provide add-ons to these frameworks that aim to simplify the setup of experiments and the analysis of results. Furthermore, it is mostly common practice that researchers make their models and architectures publicly available (model zoos), increasing visibility in their field but also facilitating their application in other studies.

### Creating representative benchmarks

Todays benchmark datasets are limited both in the number of species and in the number of images (see Table 2) due to the tremendous effort for either collecting fresh specimens and imaging them in a lab or for taking images in the field. Taking a closer look at datasets, it becomes obvious that they were created with an application in computer vision and machine learning in mind. They are typically created by only a few people acquiring specimens or images in a short period of time, from a limited area, and following a rigid procedure for their imaging. As a result, the plants of a given species in those datasets are likely to represent only a few individual plants grown closely together at the same time. Considering the high variability explained before, these datasets do not reflect realistic conditions.

Using such training data in a real-world identification application has little chance to truly classify new images collected at different periods, at different places, and acquired differently [63]. Towards real-life applications, studies should utilize more realistic images, e.g., containing multiple, overlapped, and damaged leaves and flowers. Images should have real, complex backgrounds and should be taken under different lighting conditions. Large-scale, well-annotated training datasets with representative data distribution characteristics are crucial for the training of accurate and generalizable classifiers. This is especially true for the training of Deep Convolutional Neural Networks that require extensive training data to properly tune the large set of parameters. The research community working on the ImageNet dataset [71] and the related benchmark is particularly important in this regard. ImageNet aims to provide the most comprehensive and diverse coverage of the image world. It currently contains more than 14 million images categorized according to a hierarchy of almost 22,000 English nouns. The average number of training images per category is in the range of 600 and 1,200, being considerable larger than any existing plant image collection.

First efforts have been made recently to create datasets that are specifically designed for machine learning purposes—a huge amount of information, presorted in defined categories. The PlantCLEF plant identification challenge initially provided a dataset containing 71 tree species from the French Mediterranean area depicted in 5,436 images in 2011. This dataset has grown to 113,205 pictures of herb, tree, and fern specimens belonging to 1,000 species living in France and the neighboring countries in 2016. Encyclopedia Of Life (EOL) [72], being the world's largest data centralization effort concerning multimedia data for life on earth, currently provides about 3.8 million images for 1.3 million taxa. For angiosperms, there are currently 1.26 million images, but only 68% of them are reviewed and trusted with respect to the identified taxa [73].

### Crowdsourcing training data

Upcoming trends in crowdsourcing and citizen science offer excellent opportunities to generate and continuously update large repositories of required information. Members of the public are able to contribute to scientific research projects by acquiring or processing data while having few prerequisite knowledge requirements. Crowdsourcing has benefited from Web 2.0 technologies that have enabled user-generated content and interactivity, such as wiki pages, web apps, and social media. iNaturalist and Pl@ntNET already successfully acquire data through such channels [37]. Plant image collections that acquire data through crowdsourcing and citizen science projects today often suffer from problems that prevent their effective use as training and benchmark data. First, the number of images per species in many datasets follows a **long-tail distribution**. Thousands of images are acquired for prominent taxa, while less prominent and rare taxa are represented by only a few and sometimes no images at all. The same fact applies to the number of images per organ per taxon. While prominent organs such as the flower of angiosperms are well populated, other organs such as fruits are often underrepresented or even missing. Second, collections contain a high degree of **image and tag heterogeneity**. As we elaborated in our discussion of identification challenges, the acquisition process is a main contributor of image variability. In a crowdsourcing environment, this fact is even exacerbated since contributors with very different backgrounds, motivations, and equipment contribute observations. Image collections today contain many examples not sufficient for an unambiguous identification of the displayed taxon. They may be too blurry or lack details. Collections also suffer from problems such as heterogeneous organ tags (e.g., "leaf" versus "leaves" versus "foliage"), manifold plant species synonyms used alternatively, and evolving and concurrent taxonomies. Third, nonexpert observations are more likely to contain **image and metadata noise**. Image noise refers to problems such as highly cluttered images, other plants depicted along with the intended species, and objects not belonging to the habitat (e.g., fingers or insects). Metadata noise refers to problems such as wrongly identified taxa, wrongly labeled organs, imprecise or incorrect location information, and incorrect observation time and date.

These problems show that crowdsourced content deserves more effort for maintaining sufficient data quality. An examination of a small number of randomly sampled images from the Pl@ntNET initiative and their taxa attributions indicated that misclassifications are in the range of 5% to 10%. In a first attempt to overcome these problems, Pl@ntNET introduced a star-based quality rating for each image and uses a community based review system for taxon annotations, whereas EOL offers a "trusted" tag for each taxon that has been identified within an image by an EOL curator. We argue that multimedia data should be based on common data standards and protocols, such as the Darwin Core [74], and that a rigorous review system and quality control workflows should be implemented for community based data assessment.

### Analyzing the context of observations

We argue that it is hard to develop a plant identification approach for the worlds estimated 220,000 to 420,000 angiosperms that solely relies on image data. Additional information characterizing the context of a specimen should be taken into consideration. Today, mobile devices allow for high quality images acquired in well choreographed and adaptive procedures. Through software specifically developed for these devices, users can be guided and trained in acquiring characteristic images in situ. Given that mobile devices can geolocalize themselves, acquired data can be spatially referenced with high accuracy allowing to retrieve context information, such as topographic characteristics, climate factors, soil type, land-use type, and biotope. These factors explaining the presence or absence of species are already used to predict plant distribution and should also be considered for their identification. Temporal information, i.e., the date and the time of an observation, could allow adaptation of an identification approach to species' seasonal variations. For example, the flowering period can be of high discriminative power during an identification. Furthermore, recorded observations in public repositories (e.g., Global Biodiversity Information Facility GBIF) can provide valuable hypotheses as to which species are to expect or not to expect at a given location. Finally, additional and still-emerging sensors built into mobile devices allow for measuring environmental variables, such as temperature and air pressure. The latest cameras can acquire depth maps of specimens along with an image and provide additional characteristics of an observation and its context further supporting the identification.

### From taxa-based to character-based training

In automated species identification, researchers solely aim to classify on the species level so far. An alternative approach could be classifying plant characteristics (e.g., leaf shape categories, leaf position, flower symmetry) and linking them to plant character databases such as the TRY Plant Trait Database [75] for identifying a wide range of taxa. In theory, untrained taxa could be identified by recognizing their characters. So far, it is uncertain whether automated approaches are able to generalize uniform characters from nonuniform visual information. Characters that are shared across different taxa are often differently developed per taxon, making their recognition a particular challenge.

### Utilizing the treasure of herbarium specimens

Herbaria all over the world have invested large amounts of money and time in collecting samples of plants. Rather than going into the field for taking images or for collecting specimens anew, it would be considerably less expensive to use specimens of plants that have already been identified and conserved. Today, over 3,000 herbaria in 165 countries possess over 350 million specimens, collected in all parts of the world and over several centuries [76]. Currently, many are undertaking large-scale digitization projects to improve their access and to preserve delicate samples. For example, in the USA, more than 1.8 million imaged and georeferenced vascular plant specimens are digitally archived in the iDigBio portal, a nationally funded and primary aggregator of museum specimen data [76]. This activity is likely going to be expanded over the coming decade. We can look forward to a time when there will be huge repositories of taxonomic information, represented by specimen images, accessible publicly through the internet. However, very few previous researchers utilized herbaria sheets for generating a leaf image dataset [58, 69, 77–79]. On the other hand, analyzing herbaria specimens may not be suitable for training identification approaches applied in a real environment [69]. The material is dried, and thereby, original colors change drastically. Furthermore, all herbaria specimens are imaged flattened on a plain homogeneous background, altering their structure and arrangement. In conclusion, more research on the detection and extraction of characteristics from herbaria specimens is required. It is also an open research question (how to train classifiers on herbaria specimens that are applicable on fresh specimens).

### Interdisciplinary collaborations

Twelve years ago, Gaston and O’Neill [13] argued that developing successful identification approaches requires novel collaborations between biologists and computer scientists with personnel that have significant knowledge of both biology and computing science. Interestingly, automated plant species identification is still mostly driven by academics specialized in computer vision, machine learning, and multimedia information retrieval. Very few studies were conducted by interdisciplinary groups of biologists and computer scientists in the previous decade [16]. Research should move towards more interdisciplinary endeavors. Biologists can apply machine learning methods more effectively with the help of computer scientists, and the latter are able to gain the required exhaustive understanding of the problem they are tacking by working with the former.

## A vision of automated identification in the wild

We envision identification systems that enable users to take images of specimens in the field with a mobile device's built-in camera system, which are then analyzed by an installed application to identify the taxon or to at least get a list of candidate taxa. This approach is convenient, since the identification requires no work from the user except for taking an image and browsing through the best matching species. Furthermore, minimal expert knowledge is required, which is especially important given the ongoing shortage of skilled botanists. An accurate automated identification system also enables nonexperts with only limited botanical training and expertise to contribute to the survey of the world's biodiversity. Approaching trends and technologies, such as augmented reality, data glasses, and 3D-scans, give such applications a long-term research and application perspective. Furthermore, large-character datasets can be generated automatically (for instance, by taking measurements from thousands of specimens across a single taxon). We cannot only derive more accurate descriptions of a species and its typical character expressions, but also study the statistical distribution of each character, including variance and skew. Furthermore, image processing provides the possibility to extract not only the linear measurements typical of botanical descriptions (leaf length, leaf width, petal length, etc.), but also more sophisticated and precise descriptions such as mathematical models of leaf shapes.
